# Genome Sequence of *Arthrobacter koreensis* 5J12A, a Plant Growth-Promoting and Desiccation-Tolerant Strain

**DOI:** 10.1128/genomeA.00648-15

**Published:** 2015-06-11

**Authors:** Maximino Manzanera, Juan Jesús Narváez-Reinaldo, Cristina García-Fontana, Juan Ignacio Vílchez, Jesús González-López

**Affiliations:** Institute for Water Research and Department of Microbiology, University of Granada, Granada, Spain

## Abstract

*Arthrobacter koreensis* 5J12A is a desiccation-tolerant organism isolated from the *Nerium oleander* rhizosphere. Here, we report its genome sequence, which may shed light on its role in plant growth promotion. This is believed to be the first published genome of a desiccation-tolerant plant growth promoter from the genus *Arthrobacter*.

## GENOME ANNOUNCEMENT

Plant growth-promoting bacteria (PGPB) colonize plants and enhance their growth through different mechanisms, such as by increasing nutrient bioavailability and plant bioassimilation, reducing the effects of soil plant pathogens, producing substances that enhance plant growth, and removing from the soil detrimental molecules, such as toxic compounds that can impair plant growth (1–7). Recently, several species of the genus *Arthrobacter* were described as PGPB, including *Arthrobacter pascens*, *Arthrobacter nitroguajacolicus*, *Arthrobacter nicotinovorans*, *Arthrobacter humicola, Arthrobacter woluwensis*, and *Arthrobacter protophormiae* to name a few (8–12). Among them, *Arthrobacter koreensis* 5J12A, isolated from *Nerium oleander* rhizosphere soil, was found as part of a collection of desiccation-tolerant microorganisms comprising *Arthrobacter siccitolerans* 4J27, *Rhodococcus* sp. 4J2A2, and *Leucobacter* sp. 4J7B1 (13–17).

Here, we report the whole-genome sequence of *Arthrobacter koreensis* 5J12A, obtained with pyrosequencing technology implemented in the 454 Life Science–Roche platform with a combined approach based on shotgun and 8-kb mate pair sequencing (Life-sequencing SL, Valencia, Spain) (18). With the shotgun sequencing approach, 81,219 sequences were obtained with an average read length of 627 nucleotides. With the mate pair sequencing strategy, 97,724 sequences with an average read length of 299 bases were obtained. The total number of sequenced bases was 178,943, representing a sequencing depth of around 20×. For *de novo* assembly, Newbler Assembler version 2.6 was used with default parameters. This assembly yielded 27 contigs, 13 of which were larger than 500 bp. The *N*_50_ of the contig assembly was 540,272 bp, and the largest contig was 793,085 bp. Mate pair information indicated that most of these contigs were ordered in three scaffolds, the largest comprising 3,461,065 bp. The estimated genome size of 3.8 Mb was deduced from this combination of scaffolds and contigs with an average percentage guanine + cytosine (GC) content of 64.48%. To attempt gap closure, gap-spanning clones and PCR products were used, and putative coding sequences were predicted. A pipeline implemented at Life-sequencing, and protein-coding sequences (CDS), were predicted with Glimmer (19–21), RNAmmer (22), tRNAScan (23, 24), and BLAST (25, 26) in combination for gene annotation. A total of 3,195 protein-coding genes, 5 rRNA operons, and 50 tRNA genes were found in this genome.

On the basis of this genome sequence we propose the presence of pathways for the biosynthesis of plant hormones, including auxin, salicylic acid, ethylene, abscisic acid, strigolactone, cytokinin, brassinosteroid, gibberellin, and jasmonic acid.

The complete genome sequence of *Arthrobacter koreensis* 5J12A will contribute to the development of biotechnological applications in the field of agriculture, particularly techniques to promote plant growth with bacterial inoculants (27), and in the field of anhydrobiotic engineering for the production of xeroprotectants and the conservation of cells in a dry state (28–30).

### Nucleotide sequence accession numbers.

The complete genome sequence of *Arthrobacter koreensis* 5J12A has been deposited in the ENA database under project ID PRJEB8453 and in the TBL/EMBL/GenBank databases under the accession numbers CECE01000001 to CECE01000027.
